# 505. Drug-drug Interaction Profiling of Obeldesivir, A Promising Oral Treatment for COVID-19

**DOI:** 10.1093/ofid/ofad500.574

**Published:** 2023-11-27

**Authors:** Elham Amini, Mark Shelton, Anuja Raut, Anna Kwan, Deqing Xiao, Shuguang Chen, Sharline Madera, Joe Llewellyn, Helen Winter, Rita Humeniuk

**Affiliations:** Gilead Sciences, Inc., Foster City, California; Gilead Sciences, Inc., Foster City, California; Gilead Sciences, Inc., Foster City, California; Gilead Sciences, Inc., Foster City, California; Gilead Sciences, Inc., Foster City, California; Gilead Sciences, Inc, Foster City, California; Gilead Sciences, Inc., Foster City, California; Gilead Sciences, Inc., Foster City, California; Gilead Sciences, Inc., Foster City, California; Gilead Sciences, Inc., Foster City, California

## Abstract

**Background:**

Obeldesivir (ODV), an orally administered RNA-dependent RNA polymerase inhibitor, is a GS-441524 prodrug under investigation for the treatment of COVID-19. Herein, we assessed ODV as both perpetrator and victim of DDIs in healthy adults.

**Methods:**

This Phase 1, open-label, multicenter, multicohort, fixed- or randomized-sequence crossover study evaluated ODV as a perpetrator of cytochrome P450 3A4 (CYP3A4), P-glycoprotein (P-gp) transporter, organic anion transporting polypeptide (OATP) 1B1/1B3, and organic cation transporter 1 (OCT1)-mediated DDIs and ODV as a victim of P-gp inhibition and gastric acid suppression in healthy participants (**Table 1**). Cohort size was based on predefined no-effect bounds. Plasma concentrations of GS-441524, an ODV metabolite, and probe substrates (ie, midazolam [MDZ], 1'-OH-MDZ, dabigatran [DAB], pitavastatin [PIT], and metformin [MET]), were quantified via liquid chromatography-tandem mass spectrometry. Noncompartmental analysis was performed in Phoenix WinNonlin™ to estimate pharmacokinetic (PK) parameters (C_max_, AUC_last_, and AUC_inf_). Test and reference treatments were compared using geometric least-squares mean (GLSM) ratios and two-sided 90% CIs.

**Results:**

As a perpetrator, ODV did not meaningfully affect plasma PK of MDZ, its metabolite (1'-OH-MDZ), or MET; GLSMs and 90% CIs were mostly within predefined no-effect bounds. ODV increased PIT PK parameters by 28-36% and decreased those of DAB by 22-28%. As a victim, ODV coadministration with ritonavir (RTV) did not impact plasma PK of GS-441524. The C_max_ of GS-441524 decreased by 33% with famotidine, but AUC_last_ and AUC_inf_ were within predefined no-effect bounds. Although complete safety data are not yet available, no serious adverse events, deaths, or study drug-related discontinuations were reported.

**Table 1.**
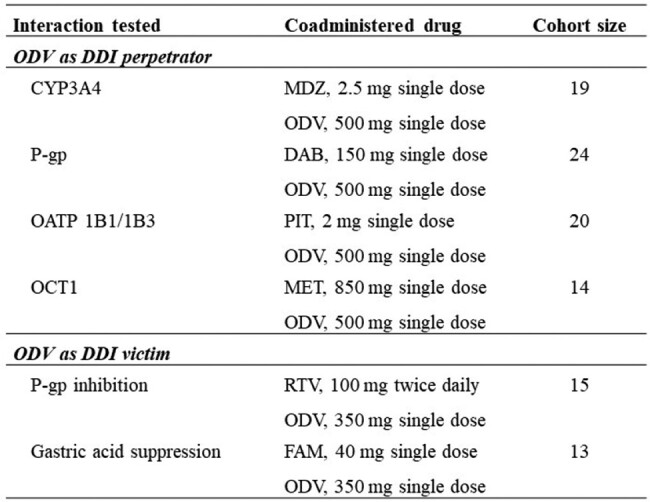
Interactions Evaluated in Clinical Study As Guided by Results of In Vitro Screening of ODV as Victim or Perpetrator for Drug-drug Interactions CYP3A4, cytochrome P450 3A4; DAB, dabigatran; FAM, famotidine; MET, metformin; MDZ, midazolam; OATP, organic anion transporting polypeptide; OCT1, organic cation transporter 1; ODV, obeldesivir; P-gp, P-glycoprotein; PIT, pitavastatin; RTV, ritonavir.

**Conclusion:**

ODV is not a clinically relevant inhibitor of CYP3A4, P-gp, OATP 1B1/1B3, or OCT1. There were no clinically significant effects of P-gp inhibition or increased gastric pH on plasma PKs of GS-441524. ODV is a promising oral treatment for COVID-19 with low potential for DDIs that can be given without regard for these concomitant medications.

**Disclosures:**

**Elham Amini, PharmD, PhD**, Gilead Sciences, Inc.: Employee|Gilead Sciences, Inc.: Stocks/Bonds **Mark Shelton, PharmD**, Certara, Inc.: Employee|Gilead Sciences, Inc.: Former Employee **Anuja Raut, MS, MS**, Gilead Sciences, Inc.: Employee|Gilead Sciences, Inc.: Stocks/Bonds **Anna Kwan, BS**, Gilead Sciences, Inc.: Employee|Gilead Sciences, Inc.: Stocks/Bonds **Deqing Xiao, PhD**, Gilead Sciences, Inc.: Employee|Gilead Sciences, Inc.: Stocks/Bonds **Shuguang Chen, PhD**, Gilead Sciences, Inc.: Employee|Gilead Sciences, Inc.: Stocks/Bonds **Sharline Madera, MD, PhD**, Gilead Sciences, Inc.: Employee|Gilead Sciences, Inc.: Stocks/Bonds **Joe Llewellyn, PharmD**, Gilead Sciences, Inc.: Employee|Gilead Sciences, Inc.: Stocks/Bonds **Helen Winter, PhD**, Gilead Sciences, Inc.: Employee|Gilead Sciences, Inc.: Stocks/Bonds **Rita Humeniuk, PhD**, Gilead Sciences, Inc.: Employee|Gilead Sciences, Inc.: Stocks/Bonds

